# HER2 in Non-Small Cell Lung Cancer: A Review of Emerging Therapies

**DOI:** 10.3390/cancers14174155

**Published:** 2022-08-27

**Authors:** Natalie F. Uy, Cristina M. Merkhofer, Christina S. Baik

**Affiliations:** Division of Medical Hematology and Oncology, Department of Medicine, University of Washington, Seattle, WA 98195, USA

**Keywords:** non-small-cell lung cancer, *HER2* amplification, *HER2* mutation, HER2 overexpression, exon 20 mutation, targeted therapies, driver mutation

## Abstract

**Simple Summary:**

There are growing data on targeting HER2 alterations, which include gene mutations, gene amplifications, and protein overexpression, for non-small cell lung cancer (NSCLC). Currently, there are limited targeted therapies approved for NSCLC patients with HER2 alterations, and this remains an unmet clinical need. There has been an influx of research on antibody–drug conjugates, monoclonal antibodies, and tyrosine kinase inhibitors. This review discusses the diagnostic challenges of HER2 alterations in NSCLC and summarizes recent progress in HER2 targeted drugs for both clinicians and researchers treating this patient population.

**Abstract:**

Human epidermal growth factor receptor 2 (HER2), a member of the ERBB family of tyrosine kinase receptors, has emerged as a therapeutic target of interest for non-small cell lung cancer (NSCLC) in recent years. Activating HER2 alterations in NSCLC include gene mutations, gene amplifications, and protein overexpression. In particular, the *HER2* exon 20 mutation is now a well clinically validated biomarker. Currently, there are limited targeted therapies approved for NSCLC patients with HER2 alterations. This remains an unmet clinical need, as HER2 alterations are present in 7–27% of de novo NSCLC and may serve as a resistance mechanism in up to 10% of *EGFR* mutated NSCLC. There has been an influx of research on antibody–drug conjugates (ADCs), monoclonal antibodies, and tyrosine kinase inhibitors (TKIs) with mixed results. The most promising therapies are ADCs (trastuzumab-deruxtecan) and novel TKIs targeting exon 20 mutations (poziotinib, mobocertinib and pyrotinib); both have resulted in meaningful anti-tumor efficacy in *HER2* mutated NSCLC. Future studies on HER2 targeted therapy will need to define the specific HER2 alteration to better select patients who will benefit, particularly for *HER2* amplification and overexpression. Given the variety of HER2 targeted drugs, sequencing of these agents and optimizing combination therapies will depend on more mature efficacy data from clinical trials and toxicity profiles. This review highlights the challenges of diagnosing HER2 alterations, summarizes recent progress in novel HER2-targeted agents, and projects next steps in advancing treatment for the thousands of patients with HER2 altered NSCLC.

## 1. Introduction

Improved understanding of oncogenic driver alterations and targeted therapy development has revolutionized the treatment of patients with non-small cell lung cancer (NSCLC). Comprehensive molecular assessment has become a standard part of NSCLC management. However, not all oncogenic driver alterations have effective targeted therapies, highlighting the need for novel therapeutic strategies.

**Figure 1:**
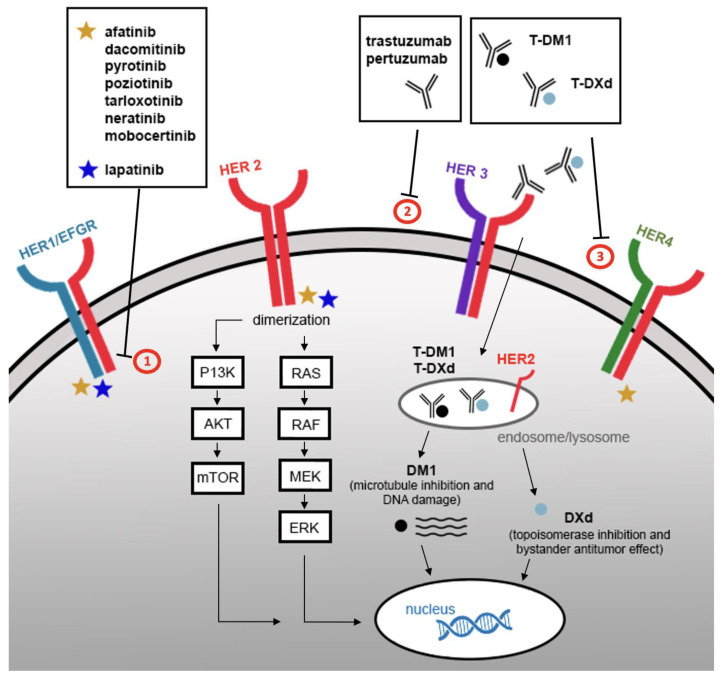
Non–small cell lung cancer HER2 tumorigenesis pathways and targeted therapy mechanisms. The HER2 extracellular domain does not have a known soluble ligand and is activated by forming homo or heterodimers, which leads to phosphorylation and activation of downstream PI3K/AKT and MEK/ERK pathways. (1) Tyrosine kinase inhibitors block phosphorylation of the tyrosine kinase residues, inhibiting cell proliferation. (2) Monoclonal antibodies bind to the extracellular domain of HER2 to block homo and heterodimerization. (3) Antibody–drug conjugates (ADC) incorporate the HER2 targeted actions of trastuzumab with a cytotoxic component (microtubule inhibitor or topoisomerase I inhibitor) connected by a cleavable tetrapeptide-based linker. Upon degradation of the HER2-ADC complex in endosomes/lysosomes, the cytotoxic component is released. This allows for selective delivery into HER2 overexpressing cells, resulting in cell cycle arrest and apoptosis.

Human epidermal growth factor receptor 2/Erb-B2 receptor tyrosine kinase 2 (HER2/*ERBB2*), a tyrosine kinase receptor in the EGFR (epidermal growth factor receptor) family, is an emerging therapeutic target [1]. HER2 does not have a known soluble ligand and downstream signaling is triggered by dimerization with other ligand-bound HER family members, which leads to phosphorylation and activation of downstream PI3K/AKT and MEK/ERK pathways. Alterations in the HER2 pathway drive oncogenesis by increased dimerization and autophosphorylation, leading to uncontrolled cell growth. Novel therapies are aimed at disrupting this pathway (Figure 1).

In this review, we aim to characterize the types of HER2 alterations in NSCLC, discuss the diagnostic challenges in identifying activating HER2 alterations, and review the application of HER2 targeted agents in patient care by discussing clinical trial data on HER2 agents.

## 2. Types of HER2 Alterations

Three main HER2 activating mechanisms have been described: gene mutations, gene amplification, and protein overexpression [2].

Oncogenic mutations in *HER2* are present in 2–4% of NSCLC and can be identified by genetic sequencing, often by next generation sequencing (NGS) in clinical settings. These mutations are predominantly found in females, never-smokers, and patients with lung adenocarcinomas [3]. The most frequent mutations are *HER2* exon 20 insertions, which have emerged as a particular area of therapeutic interest [1]. *HER2* mutations are almost always mutually exclusive with activating mutations in other oncogenic drivers such as *EGFR, KRAS, BRAF, NRAS, PIK3CA, MEK1* and *AKT*, as well as with *ALK* rearrangements [1]. *HER2* mutations are heterogeneous, which has made developing therapeutics challenging. The majority are insertions or duplications between amino acids 772 and 780, but point mutations and other insertions have also been reported [1,4]. In addition, to select *HER2* mutations serving as a primary oncogenic driver in NSCLC, these may also present as an acquired mechanism of resistance to EGFR tyrosine kinase inhibitor (TKI) treatment [5,6]. The prognostic value of *HER2* mutations remains unclear. Higher rates of brain metastases have been noted in *HER2* mutant lung cancers compared with other driver mutations [7]. Notably, the exon 20 YVMA insertion is associated with a higher incidence of brain metastases and inferior outcomes with chemotherapy [8].

*HER2* amplification occurs in 3% of NSCLC cases without prior EGFR TKI treatment and may account for up to 10% of acquired resistance to EGFR TKI therapy [9]. In contrast to *HER2* mutations, a higher proportion of *HER2* amplified patients are male smokers [10]. Amplification can be detected with fluorescence in situ hybridization (FISH). There is no standardized definition of *HER2* amplification. However, a commonly accepted definition is a ratio of ≥ 2 of the *HER2* gene copy number to centromeres [*HER2*/*chromosome enumeration probe 17 (CEP17)*] [11]. The prognostic value of *HER2* amplification is unclear, although one observational study in resected NSCLC found that high levels of *HER2* amplification (defined as *HER2*/*CEP17* ratio ≥ 5 or copy number ≥ 10) in resected NSCLC were associated with shorter overall and disease-free survival compared to lower levels [9].

HER2 protein overexpression is found in 2–20% of NSCLC, with the frequency varying depending on the level of overexpression. Overexpression is commonly assessed using immunohistochemistry (IHC). There is no consensus on the definition of HER2 protein overexpression in lung cancer, but clinical studies frequently use a score of 2+ (“weak to moderate complete membrane staining observed in >10% of tumor cells”) or 3+ (“circumferential membrane staining that is complete, intense and in >10% of tumor cells”) as per the American Society of Clinical Oncology/College of American Pathologists breast cancer guidelines [11]. HER2 overexpression is also associated with a poor prognosis, with some suggesting that this may be the case for women but not men [12,13].

Contrary to breast cancer, where HER2 overexpression often occurs concurrently with *HER2* amplification, this co-occurrence has been less consistently observed in lung cancer. There is no significant correlation between an increase in *HER2* gene copy number and protein overexpression. Mutations in the *HER2* gene are also not clearly associated with increased levels of *HER2* amplification. In fact, *HER2* amplification is often mutually exclusive from *HER2* mutations, and both have been suggested as distinct entities [1,10].

The lack of correlation between *HER2* gene mutations, gene amplification, and protein overexpression makes it challenging to define HER2 altered lung cancers. The *HER2* exon 20 insertion mutation is emerging as a clinically validated predictive biomarker, but the role of non-exon 20 mutations, amplification, and overexpression are less clear. Currently, “HER2 positivity” is often used to refer to the presence of a mutation, and more specific nomenclature and definitions are needed for amplification and overexpression, especially in clinical trial settings. Likewise, methods to detect “HER2 positivity” in NSCLC should be standardized and optimized for each type of HER2 alteration.

## 3. Treatment of HER2-Altered NSCLC

Initial clinical trials of HER2-targeted agents had disappointing results likely due to lack of patient selection [14,15] and slowed down the progress of clinical development. However, the development of novel drugs and improved patient selection has yielded an influx of studies with promising HER2-directed therapies in recent years. Antibody-drug conjugate (ADC) therapy has shown the most efficacy thus far, and several tyrosine kinase inhibitors (TKIs) are in development.

### 3.1. Antibody-Drug Conjugates

#### 3.1.1. Trastuzumab–Deruxtecan (T-DXd)

Fam-trastuzumab–deruxtecan (T-DXd) is emerging as a promising drug in *HER2* mutant NSCLC; recently it became the only FDA approved targeted therapy for NSCLC patients with *HER2* mutations. T-DXd is an ADC composed of trastuzumab, a monoclonal antibody against HER2, linked to a topoisomerase I inhibitor, deruxtecan. A recent study using lung cancer cell lines and patient-derived xenograft models showed that *HER2* mutant NSCLC models showed increased internalization of ADC therapy compared to wildtype cells, likely explaining the clinical observation of ADC efficacy in this patient population [16].

A phase I first-in-human clinical study investigated T-DXd for the treatment of patients with HER2 overexpressing (IHC ≥ 1+) or *HER2* mutant advanced non-breast, non-gastric solid tumors. Anti-tumor efficacy was observed across multiple tumor types, including HER2 expressing and *HER2* mutant NSCLC. Patients with *HER2* mutant NSCLC had increased tumor reduction relative to patients without documented mutations, regardless of HER2 overexpression status. In the subgroup of patients with NSCLC with HER2 overexpression or a *HER2* mutation, the objective response rate (ORR) was 55.6% with a median progression-free survival (PFS) of 11.3 months [17] (Table 1).

In the DESTINY-Lung01 trial, T-DXd was evaluated in two populations, overexpressing HER2 (IHC 2+ or 3+) and *HER2* mutated NSCLC. In an interim analysis of 49 HER2 overexpressing patients, the ORR was 24.5% and median PFS was 5.4 months. All patients had at least one grade ≥ 1 adverse effect (AE), most commonly nausea and decreased appetite, and grade ≥ 3 AE was reported in 73.5% of patients. One serious AE of interest was interstitial lung disease (ILD); 16.3% of patients had ILD, with 6% having grade 5 ILD [18]. In a separate study, T-DXd was administered to 91 patients with *HER2* mutated NSCLC that had progressed on standard treatment at the investigator’s discretion. The ORR was 55%, and median PFS was 8.2 months. Efficacy was noted across subgroups, including patients treated with prior HER2 TKI therapy and in patients with central nervous system (CNS) metastases. AE included gastrointestinal and hematological events, particularly neutropenia. In this cohort, 26% of patients had ILD of any grade, and 7% had grade 3–5 ILD. The 2 patients who died of ILD had previously received immunotherapy [19]. Activating *HER2* mutations are thought to enhance receptor internalization and intracellular uptake of the HER2-ADC complex, which may explain the higher efficacy of T-Dxd in patients with *HER2* mutant NSCLC versus HER2 overexpression [16].

#### 3.1.2. Ado Trastuzumab–Emtansine (T-DM1)

Ado Trastuzumab–emtansine (T-DM1) is an ADC that links trastuzumab with a cytotoxic microtubule inhibitor, DM-1. T-DM1 was evaluated as part of a phase II basket trial in 18 *HER2* mutant lung cancer patients, 11 with a *HER2* exon 20 mutation, and 2 with a concurrent *HER2* amplification. Among these patients, an ORR of 44% and a median PFS of 5 months were observed. Responses were seen across *HER2* mutation subtypes, including exon 20 insertions as well as transmembrane and extracellular domain point mutations. There was no association between IHC score and response to T-DM1. Common adverse effects of T-DM1 included liver transaminitis, thrombocytopenia, and nausea [20].

In contrast, a phase II trial of T-DM1 monotherapy in relapsed NSCLC with HER2 alterations (IHC 3+, IHC 2+ and FISH *HER2*/*CEP17* ratio ≥ 2, or exon 20 insertion mutation) was terminated early because of limited efficacy. The ORR was 6.7% and median PFS was 2.0 months [21]. A phase II trial in patients with previously treated advanced HER2 overexpressing NSCLC also demonstrated limited efficacy of T-DM1. No treatment responses were observed in the IHC 2+ cohort, although ORR was 20% in the IHC 3+ cohort [22]. A more recent T-DM1 study on only *HER2* exon 20 insertion mutated NSCLC found a numerically higher ORR (38.1%); however, PFS was only 2.8 months [23].

#### 3.1.3. Summary of Evidence regarding HER2 ADC

ADC (Table 1) have shown great clinical benefit in breast and gastric cancers, and there has been much interest in exploring their benefits in *HER2* mutant NSCLC. While results have been mixed for T-DM1, T-DXd appears promising given the high ORR and durable responses observed in the DESTINY-Lung01 trial. T-DXd was granted breakthrough therapy designation by the FDA for *HER2* mutant NSCLC; it was recently approved for advanced and metastatic NSCLC patients with *HER2* mutations after first line therapy. T-DXd will likely be a cornerstone of HER2-directed therapy in this population moving forward. There are ongoing clinical trials for T-DXd as first line monotherapy and in combination with immunotherapy and chemotherapy.

### 3.2. Monoclonal Antibodies

#### 3.2.1. Trastuzumab

Trastuzumab is a monoclonal immunoglobulin G1 humanized murine antibody that binds to the HER2 extracellular domain and inhibits dimerization. In the HOT1303-B trial, patients with HER2 altered NSCLC (IHC 2/3+ overexpression and/or mutations) previously treated with at least two lines of therapy received trastuzumab monotherapy. No patients responded to trastuzumab monotherapy in both overexpressed and mutated NSCLC [24]. In another similar study, there were no tumor responses to trastuzumab monotherapy cohort in HER2 expressing NSCLC [25] (Table 2).

Trastuzumab has also been evaluated in combination with chemotherapy in patients with NSCLC. Older studies of patients with HER2 overexpressing NSCLC treated with trastuzumab with either docetaxel [25], carboplatin and paclitaxel [26], and cisplatin and gemcitabine [27] all demonstrated mixed results, with the ORR ranging from 0% with docetaxel to 36% with cisplatin and gemcitabine. The only randomized trial in this group compared cisplatin and gemcitabine with or without trastuzumab in untreated HER2 overexpressing (defined as IHC 2+ or 3+) NSCLC patients. Efficacy was similar between the chemotherapy alone arm (ORR 41%; median PFS 7.0 months) and the combination chemotherapy and trastuzumab arm (ORR 36%; median PFS 6.1 months). However, the subset of 6 patients with HER2 IHC 3+ in the trastuzumab arm had a notably higher ORR of 83% and median PFS of 8.5 months. Both treatment arms had similar proportions of gastrointestinal and hematologic AE. Rare AE of decreased left ventricular ejection fraction was noted in the trastuzumab arm [28].

**Table 2:** Studies of Monoclonal Antibodies in HER2 altered NSCLC.

Drug	Trial	NSCLC Population (*n*)	Overall Response Rate	Median PFS (Months)	Median OS (Months)	Ref
trastuzumab	phase II (HOT1303-B)	HER2 IHC 2/3+ or mutation (*n* = 10)	0%	5.2	n/a	[24]
trastuzumab ± docetaxel	phase II	HER2 IHC 2/3+ (*n* = 13)	Trastuzumab: 0% Trastuzumab + docetaxel: 0%	n/a ^a^	5.7	[25]
trastuzumab + cisplatin/ gemcitabine	phase II	HER2 IHC 1+ or HER2 shed antigen level >15 ng/mL by ELISA (*n* = 21)	38%	36 weeks	n/a	[27]
trastuzumab + paclitaxel/ carboplatin	phase II (ECOG 2598)	HER2 IHC ≥ 1+ (*n* = 56)	24.5%	3.3	10.1	[26]
gemcitabine/cisplatin ± trastuzumab	phase II	HER2 IHC 2/3+, *HER2/CEP17* ratio ≥ 2, Serum HER2 ECD >15 ng/mL by ELISA (*n* = 101)	Control arm: 41% (50% in HER2 IHC 3+) Trastuzumab arm: 36% (83% in HER2 IHC 3+)	Control arm: 7.0 Trastuzumab arm: 6.1	Control arm: n/r Trastuzumab arm: 12.2	[28]
pertuzumab + trastuzumab + docetaxel	phase II (IFCT-1703 R2D2)	*HER2* exon 20 mutation (*n* = 45)	29%	6.8	n/a	[29]

PFS: progression free survival; OS: overall survival; IHC: immunohistochemistry; *CEP17*: chromosome enumeration probe 17; ECD: extracellular domain; n/a: not available; n/r: not reached. ^a^ Event free survival was 4.3 months.

#### 3.2.2. Pertuzumab

Pertuzumab is a humanized monoclonal anti-HER2 antibody that binds HER2′s dimerization domain and inhibits HER2 signaling. Pertuzumab monotherapy was evaluated in 43 patients with previously treated, unselected NSCLC, and no responses were seen. However, this poor response may be related to a lack of selection for HER2 status [14].

The IFCT-1703 R2D2 trial evaluated the combination of pertuzumab, trastuzumab, and docetaxel in patients with *HER2* mutated NSCLC after progression through platinum-based chemotherapy. The ORR was 29% and the median PFS was 6.8 months. The most frequent grade 3 AE were neutropenia, diarrhea, and anemia [29].

#### 3.2.3. Summary of Evidence regarding HER2 Monoclonal Antibodies

While HER2 monoclonal antibodies (Table 2) have benefited patients with advanced HER2 breast and gastric cancers, results in NSCLC have been more limited. In contrast to ADCs, monoclonal antibody monotherapy has not demonstrated objective tumor responses. Monoclonal antibody and chemotherapy combination regimens have resulted in numerically higher ORR. However, further data comparing combination therapy versus chemotherapy alone are needed to define whether there is a role for monoclonal antibody-based regimens in select patients with HER2 altered NSCLC.

### 3.3. Tyrosine Kinase Inhibitors

A number of TKIs have been developed to target distinct ERBB family members. Dual EGFR/HER2 TKIs (lapatinib) and pan-HER inhibitors (afatinib, neratinib, dacomitinib, pyrotinib, poziotinib, tarloxotinib, and mobocertinib) which irreversibly bind to EGFR/HER1, HER2, and HER4 tyrosine kinases have been under active investigation (Figure 1). We have highlighted TKIs that specifically included patients with *HER2* exon 20 mutant NSCLC (Table 3).

#### 3.3.1. Pyrotinib

Preclinical and phase I clinical data from breast cancer indicate that pyrotinib can irreversibly inhibit multiple HER receptors (EGFR/HER1, HER2, and HER4) and HER2 overexpressing cells in vitro and in vivo. Studies have shown a 19–53% ORR and a median PFS of 5–6 months in pretreated *HER2* mutant NSCLC, even in patients that received prior HER2 directed therapy [30,31,32]. *HER2* mutant patients with non-exon 20 mutations had an ORR comparable to exon 20 mutations [32]. Pyrotinib has also been studied in in *HER2* amplified NSCLC, demonstrating an ORR of 22.2%, and PFS of 6.3 months. The presence of other mutations (*HER2*, *EGFR*, or *TP53)* was not associated with ORR, PFS, or OS in *HER2* amplified NSCLC [33]. AEs from pyrotinib included diarrhea, elevated blood creatinine, vomiting, and anemia [31,32]. Pyrotinib is a promising agent for *HER2* mutant NSCLC and further investigation is in progress in clinical trials.

#### 3.3.2. Poziotinib

Poziotinib is an irreversible EGFR/HER1, HER2, and HER4 receptor inhibitor. Its smaller size and more flexible structure help circumvent steric hindrance in the drug-binding pocket from *HER2* exon 20 insertions. In preclinical studies using in vitro and patient-derived xenograft models of *EGFR/HER2* exon 20 mutant NSCLC, poziotinib demonstrated the most potent activity against *HER2* exon 20 mutations compared to other TKIs (erlotinib, afatinib, dacomitinib, neratinib, osimertinib, AZ5104, pyrotinib, lapatinib, and irbinitinib) [41,42]. A phase II trial evaluated poziotinib in metastatic NSCLC with *HER2* exon 20 insertion mutations, and the ORR was 27% with a median PFS of 5.5 months [34]. The ZENITH20 trial was designed to evaluate poziotinib in a large, prospective multi-cohort study (*n* = 603), which included a subgroup of 90 pretreated patients with *HER2* exon 20 mutations. An ORR of 28% was observed, with a median PFS of 5.5 months. The most common AE reported were rash, diarrhea, and stomatitis, and, notably, toxicity was not increased in patients receiving sequential immune checkpoint inhibitors (ICI) and poziotinib [35]. Poziotinib is under active investigation for the *HER2* exon 20 population in clinical trials (Table 4).

#### 3.3.3. Mobocertinib

Mobocertinib is irreversible EGFR/HER1, HER2, and HER4 receptor inhibitor with a higher affinity for *EGFR* exon 20 insertions due to a covalent bond with cysteine 797 in EGFR. It is FDA approved in NSCLC patients with *EGFR* exon 20 insertions, and has demonstrated anti-tumor activity in *HER2* exon 20 insertion mutants in pre-clinical models [43]. It was particularly effective in *HER2* exon 20 G776 > VC tumors, and synergistic with T–DM1 on *HER2* exon 20 YVMA tumors for both first–line and second–line settings after acquired resistance [44]. This agent is being actively investigated in *HER2* exon 20 mutated NSCLC, and clinical efficacy data has not yet been reported [45].

#### 3.3.4. Tarloxotinib

Tarloxotinib is designed as a prodrug that releases the activated pan-HER inhibitor, tarloxotinib-effector, under hypoxic conditions in tumors [46]. The RAIN-701 trial evaluated tarloxotinib in NSCLC patients with an *EGFR* exon 20 insertions or *HER2* activating mutations after platinum-based chemotherapy, and in any solid tumors with an *NRG1*, *EGFR*, *HER2* or *HER4* fusion. Among the 9 evaluated patients with *HER2* mutation, an ORR of 22% was observed. This agent is no longer being developed as monotherapy [36].

#### 3.3.5. Afatinib

Afatinib is an irreversible EGFR/HER1, HER2, and HER4 receptor inhibitor. While afatinib is approved in metastatic NSCLC with activating *EGFR* mutations, it has shown disappointing efficacy in *HER2* mutated NSCLC. [47] While afatinib showed promise in preclinical studies [47], afatinib monotherapy in 13 patients with *HER2* exon 20 mutated NSCLC after platinum-based chemotherapy had only an 8% ORR and a median PFS of 15.9 weeks in the NICHE trial [37]. Similarly, an ORR of 0% and a median PFS of 17 weeks were observed in 7 patients with *HER2* exon 20 mutated NSCLC receiving afatinib monotherapy. There was one response out of three *HER2* exon 20 mutant NSCLC patients receiving combination afatinib and paclitaxel [38], with AE including diarrhea, vomiting, abdominal pain. There are no strong data supporting afatinib monotherapy for the treatment of *HER2* exon 20 mutant NSCLC, and the data on afatinib combination therapy is limited.

#### 3.3.6. Neratinib

Neratinib is an irreversible EGFR/HER1, HER2, and HER4 receptor inhibitor. Neratinib showed good antitumor activity against multiple *HER2* mutations in preclinical studies; the addition of an mTOR inhibitor to a HER2 inhibitor resulted in synergistic tumor growth inhibition in breast and lung cancer cell line and mouse models [48,49]. However, this was not replicated in human studies, and neratinib monotherapy led to no objective responses in *HER2* mutant NSCLC patients [39,50]. A modest response was observed with the combination of neratinib and temsirolimus, with an ORR of 19% seen in the PUMA-NER-4201 trial selected for *HER2* exon 20 mutations [39]. The primary AE of neratinib in combination with temsirolimus were diarrhea, nausea, and increased stomatitis.

#### 3.3.7. Dacomitinib

Dacomitinib is an oral TKI that irreversibly inhibits the EGFR/HER1, HER2, and HER4, and has been shown to have promising efficacy in EGFR lung cancer studies [51]. Twenty-six NSCLC patients with *HER2* exon 20 mutations and 4 patients with *HER2* amplifications were treated with dacomitinib in a multicenter phase II trial. The ORR was 12% and 0% in *HER2* exon 20 mutant and *HER2* amplified patients, respectively, [40].

#### 3.3.8. Summary of Evidence regarding HER2 TKIs

Overall, while there has been progress on TKIs for HER2 altered NSCLC, patient numbers are low and clinical efficacy appears modest relative to TKIs targeting other NSCLC oncogenes (ie EGFR, ALK, ROS1). Less selective HER2 TKIs, including afatinib, neratinib, and dacomitinib, have had disappointing results with low or no objective tumor response. More selective HER2 TKIs, such as poziotinib, mobocertinib and pyrotinib, have demonstrated a better affinity for *EGFR* and *HER2* exon 20 variants. Accordingly, these agents have shown ORR in the 22–28% range and are being actively studied. Further work is needed to improve upon the clinical efficacy and toxicity management for HER2 TKIs. Similar to ADCs, the more selective HER2 TKIs show promise in becoming a cornerstone of HER2-directed therapy in the coming years.

### 3.4. Immunotherapy

While immune checkpoint inhibitors (ICI) are a key part of NSCLC management, retrospective data have shown limited benefit of ICI in NSCLC patients with actionable oncogenic alterations. Hypotheses for poor response to ICIs in lung cancers with driver mutations such as EGFR are a lower tumor mutation burden and a “cold”, immunosuppressive tumor microenvironment [52].

One retrospective study evaluating ICI in 551 patients with NSCLC with various oncogenic alterations included 29 patients had *HER2* exon 20 activating mutations. The ORR was 19% for all patients with oncogenic drivers, and it was 7% for patients with *HER2* mutated NSCLC. Among the patients with *HER2* mutated NSCLC, the median PFS was 2.1 months [53].

A similar retrospective study of 122 patients with *HER2* mutated NSCLC included 26 patients that were treated with ICI. PD-L1 expression was <1% in 67 patients (77%), 1–49% in 9 patients (10%), and ≥50% in 11 patients (13%). Relative to a cohort of NSCLC patients that were not biomarker-selected, PD-L1 expression was lower among patients with *HER2* mutations. The ORR to ICI was 12%, and these responses were observed in patients with *HER2* non-exon 20 (non-*HER2* YVMA) mutations. In these three patients, the median PFS was 1.9 months and the median OS was 10.4 months [54].

Another retrospective multicenter study analyzed clinical characteristics and outcomes among patients with *BRAF-, HER2-, MET-,* or *RET*-mutated NSCLC treated with ICI. Twenty-three out of 107 patients had *HER2* exon 20 mutations. Median PFS and OS were 4.7 months and 16.2 months, respectively, for the entire cohort. Among patients with *HER2* mutated disease, the ORR was 27% (6 PR), median PFS was 2.2 months, and median OS was 20.4 months [55].

These studies indicate that patients with *HER2* mutated NSCLC have limited benefit from ICI monotherapy, possibly due to similar underlying biology as other tumors with driver oncogene. Patients with *HER2* mutated NSCLC are more likely to be never smokers [3], thus may have a “cold” tumor immune microenvironment. An analysis of TCGA (The Cancer Genome Atlas) evaluated associations between *HER2* mutated solid tumors and immune features. This study showed that *HER2* mutation was associated with microsatellite instability, tumor mutation burden, and improved response to immune checkpoint inhibitor therapy [56]. In contrast, in a recent study in *EGFR* and *HER2* exon 20 insertion mutated patient NSCLC tumors, the average tumor mutational burden was 3.3 mutations/Mb and response to anti-PD1 blockade therapy was low [57]. This suggests that the immune biology of *HER2* mutated tumors are likely heterogeneous across different tumor types and *HER2* mutated NSCLC may have a similar biology as that observed for *EGFR* mutated NSCLC, although this needs to be elucidated further.

Given the limited clinical benefit of ICI in *HER2* mutated NSCLC, targeted therapies and chemotherapy should be considered for patients before considering immunotherapy as a single agent. The additive benefit of ICI in combination with chemotherapy in *HER2* mutated NSCLC is likely low although this has not been established in clinical trials. Preclinical data in mouse models suggest that combination therapy with T-DXd and anti-PD1 antibody may be more effective than either monotherapy [58]. Since drugs such as T-DXd might increase T-cell activity and upregulate PD-L1 expression, ICI in combination with HER2 therapy remains a future area of investigation.

### 3.5. Cytotoxic Chemotherapy

Data on the use of cytotoxic chemotherapy, specifically in HER2-altered NSCLC are limited. Wang et al. found that *HER2* mutated NSCLC has inferior outcomes with first line pemetrexed based chemotherapy relative to NSCLC with other oncogenic driver mutations, with reduced PFS compared with ALK/ROS1-rearranged patients (5.1 months vs. 9.2 months, *p*  =  0.004). Furthermore, there was a trend toward reduced PFS with exon 20 A775_G776insYVMA mutation compared to other *HER2* variants (4.2 vs. 7.2 months, *p*  =  0.085) [59]. In a retrospective study, Cappuzzo et al. tested tumor samples from 184 NSCLC patients for *HER2* gene copy number (28.8% HER2 by FISH) and found that *HER2* gene copy number was not associated with response to first line chemotherapy. However, this analysis was based on post hoc testing of tumor samples, and this study pre-dated the incorporation of pemetrexed-based chemotherapy regimens into the treatment of non-squamous NSCLC [60]. The Cancer and Leukemia Group B evaluated if expression of HER2 was predictive of response to chemotherapy or survival in unresectable NSCLC patients in a retrospective study; HER2 overexpression did not predict response to chemotherapy or survival [61]. Finally, a study evaluated the treatment outcomes of 44 patients with *HER2* mutated NSCLC and observed that first line pemetrexed based chemotherapy led to numerically longer progression-free survival (5.9 vs. 4.6 months, not statistically significant) compared to HER2-TKI therapy, although overall survival was numerically longer in patients who received HER2 directed therapy as first line (10.8 vs. 9.8 months, not statistically significant) [62].

Overall, these data suggest that *HER2* exon 20 mutated NSCLC may be less responsive to chemotherapy relative to NSCLC with other driver mutations, however, whether a HER2 directed therapy will be superior to chemotherapy remains unanswered and will need to be investigated in future studies.

## 4. Practical Treatment Considerations

As more data on different HER2 alterations emerge, the term “HER2-positive” is no longer sufficient. To clarify which patients respond to therapy, patients in HER2 targeted therapy studies should be defined by the specific HER2 alteration [10]. In particular, the *HER2* exon 20 insertion mutant population represents a significant unmet clinical need in NSCLC. A consensus for HER2 alteration testing in NSCLC by experts from academic centers in the United States, China, Japan, and South Korea was recently published as an effort to improve standardization [63].

Upfront *HER2* mutation testing, preferentially sequencing for exon 20 mutations, has been suggested as part of a larger routine testing panel using NGS. Since diagnostics and treatment in *HER2* amplification and overexpression is limited, routine testing in all NSCLC is not recommended. However, *HER2* amplification testing can be considered to guide treatment, such as in cases of acquired resistance to EGFR TKI therapy [63]. The practical challenges of obtaining sufficient tissue from patients can limit the ability to test for the growing list of actionable genomic alterations, including the heterogeneity of HER2 alterations. Liquid biopsies can be a useful alternative when tissue is insufficient. A wide variety of *HER2* mutations, including exon 20 insertions, have been successfully detected in ctDNA from a large series of >8000 NSCLC liquid biopsy cases [64].

The influx of investigational HER2-directed agents has been particularly robust in the last decade, yet, currently, there is only one FDA-approved HER2-targeted therapy for NSCLC. Platinum-based chemotherapy remains the preferred first line of therapy at this time. T-Dxd was just recently approved for patients with advanced and metastatic HER2 mutant NSCLC who have received prior systemic therapy. Patients should consider enrolling in HER2 targeted therapy clinical trials during any line of treatment when possible. In the future, as more ADCs and TKIs are approved by regulatory bodies, optimal sequencing of these agents will depend on more mature efficacy data from clinical trials and toxicity profiles. Where immunotherapy should be sequenced in treatment remains unclear, and likely will depend on the timing of HER2-directed therapies. Furthermore, the safety of HER2 ADCs and TKIs after immunotherapy is unknown at this time. Therefore, the use of immunotherapy likely will need to be reserved for later lines of therapy.

An important challenge in the administration of HER2 agents is the potentially significant cutaneous and GI toxicities from EGFR inhibition in HER2-targeted therapies. For example, poziotinib was associated with high proportions of grade ≥ 3 rash (48.9%) and diarrhea (25.6%). All 25 patients who achieved PR on the trial required a dose interruption and 22 required a dose reduction [35]. Pyrotinib was also associated with any grade diarrhea and anemia in 85.9% and 35.9% of patients [33]. In patients with *EGFR* exon 20 mutated NSCLC receiving mobocertinib, 83% had diarrhea of any grade, and 21% of all patients had grade ≥ 3 diarrhea [45]. For T-DXd, ILD has been reported as a rare but serious AE, particularly in patients who received immunotherapy prior [19]. This indicates the importance of proactive side effect management during HER2 targeted therapy.

## 5. Future Directions

Initial outcomes of HER2-directed therapies have been disappointing, likely due to lack of appropriate patient selection in clinical trials, lack of target selectivity in case of TKI therapies such as afatinib and lack of mechanistic understanding for antibody-based therapies in HER2 altered NSCLC. However, significant strides have been made in the past few years with the recognition of the *HER2* exon 20 insertion mutation as an oncogenic driver mutation and improved patient selection for this biomarker in clinical trials. Promising therapies have now emerged, notably the newly FDA approved trastuzumab deruxtecan, as well as poziotinib and pyrotinib. Future and ongoing studies aim to obtain mature efficacy and toxicity data of these agents and to clarify their role in the overall treatment journey of a patient. For example, DESTINY-Lung04 (NCT05048797) is a phase 3 study that compares trastuzumab deruxtecan to platinum-based chemotherapy in the first line setting. The primary endpoint is PFS, and the results of this study will provide data on the key question of optimal first line therapy in this patient population. Similarly, PINNACLE (NCT05378763) is a phase 3 study that compares poziotinib to docetaxel in patients who have had prior systemic therapy, with PFS as the primary endpoint. This study will address the role of poziotinib after initial treatment with platinum-based chemotherapy.

Additionally, several clinical trials are actively investigating novel TKIs such as BDTX-189, DZD9008, AST2818 and BAY2927088 (Table 4). This next generation of TKIs are designed to inhibit ErbB mutations while sparing ErbB wildtype, with the goal of optimizing anti-tumor efficacy without excessive toxicities [65,66].

Many opportunities remain in the field of HER2 altered NSCLC. Therapeutic investigation into HER2 over-expressed and amplified NSCLC is in its early stage and there is a need for a more in-depth investigation into their oncogenic biology, as well as better defining biomarkers to allow further investigation in clinical trials. The role of immunotherapy in HER2 altered NSCLC remains unclear and while there is unlikely to be a clinical trial of immunotherapy specifically in this patient population, a better understanding of the tumor immune microenvironment in HER2 altered NSCLC could allow for more rational use of these agents in this patient population.

HER2 alterations are now recognized as important oncogenic alterations in NSCLC. While *HER2* amplification and overexpression are less defined in NSCLC, the *HER2* exon 20 mutation is now a well clinically validated biomarker. Novel TKIs and ADC-based therapies offer higher response rates and improved survival in HER2 altered NSCLC; these therapeutic breakthroughs and increasing understanding of HER2 pathways bring hope for this challenging disease.

## Figures and Tables

**Table 1:** Studies of Antibody Drug Conjugates in HER2 altered NSCLC.

Drug	Trial	Tumor Types	NSCLC Population (*n*)	Overall Response Rate	Median PFS (Months)	Median OS (Months)	Ref
T-DXd	phase I	NSCLC, colorectal, salivary gland, breast, esophageal, endometrial, Paget’s disease, biliary tract, pancreatic, cervical, extraskeletal myxoid chondrosarcoma, small intestine adenocarcinoma	HER2 IHC ≥ 1+ or *HER2* mutation (NSCLC *n* = 18; exon 20 NSCLC *n* = 8)	Overall NSCLC: 55.6% *HER2* mutant: 72.7%	Overall NSCLC: 11.3 *HER2* mutant: 11.3	Overall NSCLC: n/r *HER2* mutant: 17.3	[17]
T-Dxd	phase II (DESTINY-Lung01)	NSCLC	HER2 IHC 2/3+ (*n* = 49)	24.5%	5.4	n/a	[18]
T-DXd	phase II (DESTINY-Lung01)	NSCLC	*HER2* mutation (*n* = 91; exon 20 = 78)	55%	8.2	17.8	[19]
T-DM1	phase II	NSCLC	*HER2* mutation (*n* = 18; exon 20 = 11)	44%	5.0	n/a	[20]
T-DM1	phase II	NSCLC	HER2 (IHC 3+, IHC 2+ and FISH *HER2*/*CEP17* ratio ≥ 2, or exon 20 mutation) (*n* = 15, IHC/FISH+ *n* = 8, exon 20 *n* = 7)	Overall: 6.7% IHC/FISH-positive: 0% Exon 20: 14.3%	2.0	10.9	[21]
T-DM1	phase II	NSCLC	HER2 IHC 2/3+ (*n* = 49)	IHC 2+: 0% IHC 3+: 20%	IHC 2+: 2.6 IHC 3+: 2.7	IHC 2+: 12.2 IHC 3+: 15.3	[22]
T-DM1	phase II	NSCLC	*HER2* exon 20 mutation (*n* = 22)	38.1%	2.8	8.1	[23]

PFS: progression free survival; OS: overall survival; T-DM1: Trastuzumab Emtansine; T-DXd: Trastuzumab Deruxtecan; IHC: immunohistochemistry; *CEP17*: chromosome enumeration probe 17; n/a: not available; n/r: not reached.

**Table 3:** Studies of Tyrosine Kinase Inhibitors in *HER2* exon 20 mutation NSCLC.

Drug	Trial	NSCLC population (*n*)	Overall Response Rate	Median PFS (Months)	Median OS (Months)	Ref
pyrotinib	phase II	*HER2* exon 20 mutation (*n* = 15)	53.3%	6.4	n/a	[30]
pyrotinib	phase II	*HER2* mutation (*n* = 60; *HER2* exon 20 mutation *n* = 56)	30%	6.9	14.4	[31]
pyrotinib	phase II	*HER2* mutation (*n* = 78, *HER2* exon 20 mutation = 62)	19.2%	5.6	10.5	[32]
pyrotinib	phase II	*HER2* amplification (*n* = 27)	22.2%	6.3	12.5	[33]
poziotinib	phase II	*HER2* exon 20 mutation (*n* = 30)	27%	5.5	15	[34]
poziotinib	phase II (ZENITH20)	*HER2* exon 20 mutation (*n* = 90)	27.8%	5.5	n/a	[35]
tarloxotinib	phase II (RAIN-701)	*EGFR* Exon 20 insertion, *HER2* activating mutation, or any solid tumors with *NRG1, EGFR, HER2* or *HER4* fusion (*n* = 23; *HER2 n* = 11)	HER2 cohort: 22%	n/a	n/a	[36]
afatinib	phase II (NICHE)	*HER2* exon 20 mutation(*n* = 13)	8%	15.9 weeks	56.0 weeks	[37]
afatinib ± paclitaxel	phase II	EGFR and HER2 (*n* = 41; *HER2* exon 20 mutation *n* = 7)	afatinib HER2: 0% afatinib + paclitaxel HER2: 33.3%	afatinib HER2: 17 weeks afatinib + paclitaxel (EGFR and HER2): 6.7 weeks	n /a	[38]
neratinib ± TEM	phase II (PUMA-NER-4201)	*HER2* exon 20 mutation (*n* = 60)	neratinib: 0% neratinib + TEM: 19%	neratinib: 3.0 neratinib + TEM: 4.1	neratinib: 10.0 neratinib + TEM: 15.8	[39]
dacomitinib	phase II	*HER2* exon 20 mutation (*n* = 26) or amplification (*n* = 4)	*HER2* exon 20 mutation: 12% *HER2* amplification: 0%	*HER2* exon 20 mutation: 3.0 *HER2* amplification: n/a ^a^	*HER2* exon 20 mutation: 9.0 *HER2* amplification: n/a ^a^	[40]

PFS: progression free survival; OS: overall survival; IHC: immunohistochemistry; n/a: not available; TEM: temsirolimus. ^a^ Range for PFS was 1–5 months, and range for OS was 5–22 months.

**Table 4:** Select Ongoing Clinical Trials in *HER2* exon 20 mutation NSCLC patients.VI. Conclusion.

Drug	Mechanism of Action	Development Phase	Sponsor	NCT Number
Trastuzumab Deruxtecan	ADC	phase III (DESTINY-Lung04)	AstraZeneca	NCT05048797
Pertuzumab + Trastuzumab + Docetaxel	Monoclonal antibody	phase II	Intergroupe Francophone de Cancerologie Thoracique	NCT03845270
Pyrotinib	TKI	phase III (PYRAMID-1)	Jiangsu HengRui Medicine Co	NCT04447118
Pyrotinib + Thalidomide	TKI	phase II	Shanghai Chest Hospital	NCT04382300
Pyrotinib	TKI	phase II	Tianjin Medical University Cancer Institute and Hospital	NCT04063462
Poziotinib	TKI	phase III (PINNACLE)	Spectrum Pharmaceuticals	NCT05378763
Poziotinib	TKI	phase II	MD Anderson Cancer Center	NCT03066206
Poziotinib	TKI	phase II	Spectrum Pharmaceuticals	NCT03318939
Mobocertinib	TKI	phase I/II	Takeda	NCT02716116
BDTX-189 (tuxobertinib)	TKI	phase I/II(MasterKey-01)	Black Diamond Therapeutics	NCT04209465
DZD9008 (sunvozertinib)	TKI	phase I/II (WU-KONG1)	Dizal Pharmaceuticals	NCT03974022
AST2818 (furmonertinib)	TKI	phase I	ArriVent BioPharma	NCT05364073
BAY2927088	TKI	phase I	Bayer	NCT05099172
